# Using Deep-Learning-Based Artificial Intelligence Technique to Automatically Evaluate the Collateral Status of Multiphase CTA in Acute Ischemic Stroke

**DOI:** 10.3390/tomography9020052

**Published:** 2023-03-16

**Authors:** Chun-Chao Huang, Hsin-Fan Chiang, Cheng-Chih Hsieh, Chao-Liang Chou, Zong-Yi Jhou, Ting-Yi Hou, Jin-Siang Shaw

**Affiliations:** 1 Department of Radiology, MacKay Memorial Hospital, Taipei 104217, Taiwan; hcc.5306@mmh.org.tw (C.-C.H.);; 2 Department of Medicine, MacKay Medical College, New Taipei City 252005, Taiwan; 3 Mackay Junior College of Medicine, Nursing, and Management, Taipei 112021, Taiwan; 4 Department of Neurology, MacKay Memorial Hospital, Taipei 104217, Taiwan; 5 Institute of Mechatronic Engineering, National Taipei University of Technology, Taipei 106344, Taiwan

**Keywords:** multiphase CTA, collateral status, artificial intelligence, convolutional neural network, acute ischemic stroke

## Abstract

Background: Collateral status is an important predictor for the outcome of acute ischemic stroke with large vessel occlusion. Multiphase computed-tomography angiography (mCTA) is useful to evaluate the collateral status, but visual evaluation of this examination is time-consuming. This study aims to use an artificial intelligence (AI) technique to develop an automatic AI prediction model for the collateral status of mCTA. Methods: This retrospective study enrolled subjects with acute ischemic stroke receiving endovascular thrombectomy between January 2015 and June 2020 in a tertiary referral hospital. The demographic data and images of mCTA were collected. The collateral status of all mCTA was visually evaluated. Images at the basal ganglion and supraganglion levels of mCTA were selected to produce AI models using the convolutional neural network (CNN) technique to automatically predict the collateral status of mCTA. Results: A total of 82 subjects were enrolled. There were 57 cases randomly selected for the training group and 25 cases for the validation group. In the training group, there were 40 cases with a positive collateral result (good or intermediate) and 17 cases with a negative collateral result (poor). In the validation group, there were 21 cases with a positive collateral result and 4 cases with a negative collateral result. During training for the CNN prediction model, the accuracy of the training group could reach 0.999 ± 0.015, whereas the prediction model had a performance of 0.746 ± 0.008 accuracy on the validation group. The area under the ROC curve was 0.7. Conclusions: This study suggests that the application of the AI model derived from mCTA images to automatically evaluate the collateral status is feasible.

## 1. Introduction

Stroke is a complex disease and is one of the major causes of long-term disability and death. Because of aggressive treatment of underlying risk factors, such as hypertension and dyslipidemia, the mortality due to stroke declines [1]. In acute ischemic stroke with large vessel occlusion, endovascular thrombectomy (EVT) has been proved to be beneficial and is able to improve functional outcome. Roughly 46.0% of patients receiving EVT can achieve a modified Rankin scale (mRS) score of 0 to 2 within 3 months, as compared with only 26.5% of patients not receiving this procedure [2]. The two basic criteria to select candidates for EVT are the time window and image evidence of large vessel occlusion. Collateral status is an additional important imaging result to identify good candidates for EVT, especially at the borderline or in an extended time window. Therefore, pre-procedural evaluation, including imaging studies, should be completed as soon as possible.

Multiphase computed-tomography angiography (mCTA) is a useful imaging tool to identify good candidates for EVT. mCTA includes an arterial phase and 2 subsequent venous phases, also known as 2 delayed arterial phases, and there will be three post-contrast images in mCTA. As compared with traditional single-phase CTA, mCTA is advantageous in the detection of arterial occlusion, with higher interrater reliability and in the evaluation of collateral status [3]. Another useful tool is computed-tomography perfusion (CTP), which can calculate the volumes of infarction core and ischemic penumbra, and these measurements can be automatically performed by some automated software [4]. These two techniques are both widely used in acute ischemic stroke. The strengths of CTP include higher correlation with clinical outcome and availability of automated postprocessing software. In contrast, mCTA does not require postprocessing software and is recommended by guidelines. However, the weak point of mCTA is that the evaluation relies on visual assessment, which might still compromise interrater and intrarater agreement and require additional time to evaluate the imaging results [5]. The management of acute ischemic stroke is as soon as possible to reverse blood flow for the ischemic brain in order to rescue these salvageable brain regions. If the results of mCTA could be evaluated automatically as CTP, the pre-EVT evaluation could be more efficient, which would be significantly beneficial for patients.

Artificial intelligence (AI) dramatically improves the performance of clinical radiology. The basic clinical application of AI is computer-aided detection, such as lesion detection in chest plain films or mammography [6,7]. The advanced clinical application includes disease classification and grading and prediction of prognosis [8,9,10]. Automatic calculation of the results of CTP is also a typical demonstration of the AI technique. If the AI technique can be used to automatically measure the collateral status of mCTA, the weak points of using mCTA in acute ischemic stroke, including time-consuming visual evaluation and discrepancy of interrater or intrarater agreement, might be solved.

This study aims to use the AI technique to develop an AI model, which can automatically evaluate the collateral status of mCTA in acute ischemic stroke with large vessel occlusion.

## 2. Methods

### 2.1. Subjects

This was a retrospective study, which had been approved by the Institutional Review Board in Mackay Memorial Hospital (20MMHIS361e). Subjects who had acute ischemic stroke with large vessel occlusion and received EVT between January 2015 and June 2020 were enrolled in this study. Other inclusion criteria included age at least 20 years old, a National Institute of Health Stroke Scale (NIHSS) at onset time of at least 8 scores, having mCTA images, having mRS at 3 months after the procedure of EVT, and having an Alberta Stroke Program Early CT Score (ASPECTS) of at least 5 points on the initial nonenhanced brain CT images. Exclusion criteria were loss of required clinical data, marked imaging motion artifact, severe underlying diseases such as malignancy, severe trauma, and aortic dissection, and acute ischemic infarction due to posterior circulation, including occlusion of vertebral artery or basilar artery.

### 2.2. Imaging Studies

**Figure 1:**
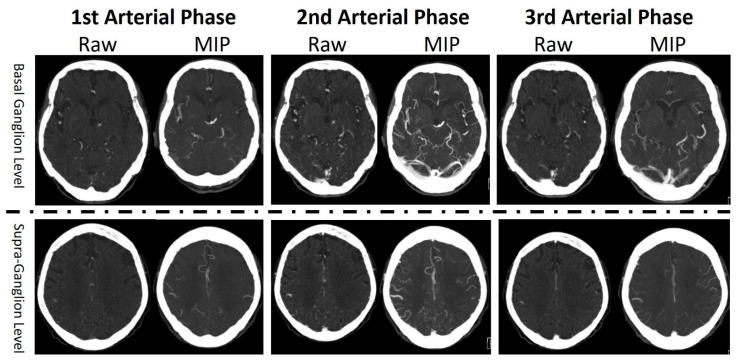
Demonstration of 12 images selected from each multiphase CTA study.

In the enrolled subjects, all the mCTA of these subjects were reviewed and graded by an experienced neuroradiologist. The collateral status of mCTA was graded based on the original concept of the development of mCTA [11]. The pial arterial filling score within the symptomatic ischemic territory using mCTA ranged from 0 to 5. A score of 5 meant good collateral status, 4 meant intermediate collateral status, and 0 to 3 meant poor collateral status. In a complete mCTA scan, there were a nonenhanced CT scan from head to neck, an arterial phase CT scan from head to neck, an 8 s delay arterial phase CT scan for the whole head, and a 16 s delay arterial phase CT scan for the whole head. The raw images were 1.5 mm on a Siemens SOMATOM Definition AS CT Scanner (Siemens Healthcare GmbH, Erlangen, Germany) and 1 mm on a Canon Aquilion PRIME CT Scanner (Toshiba Medical Systems, Nasu, Japan). Reconstruction with the maximum intensity projection (MIP) technique was applied on the first arterial phase CT images to produce axial, coronal and sagittal MIP images, as well as on the second and third arterial phase CT images, to produce axial MIP images. The settings of MIP were a 20 mm slice thickness with a 10 mm increment for axial reconstructed MIP images and a 10 mm slice thickness with a 4 mm increment for coronal and sagittal reconstructed MIP images. Two levels of the axial images were identified as the basal ganglion level and the supraganglion level. The basal ganglion level was the image when the size of the basal ganglion was visually measured at the maximum size. The supraganglion level meant the first image level when both lateral ventricles just disappeared on the axial images examined in a caudal to cranial sequence. There were 12 images selected from the mCTA study of each subject for further analysis using the AI technique. The 12 images included the raw image and the MIP image at the basal ganglion level and the supraganglion level from all three arterial phase images (Figure 1).

### 2.3. Data Preprocessing and Normalization

In this study, we mainly used Keras and Scikit-learn libraries in Python to perform data preprocessing, normalization and model establishment. Before establishing deep learning models, data preprocessing and normalization were conducted. The data in this study were images, so the data preprocessing and normalization included the denoising and adjusting contrast of images and resizing images to be identical in size.

### 2.4. Convolutional Neural Network

**Figure 2:**
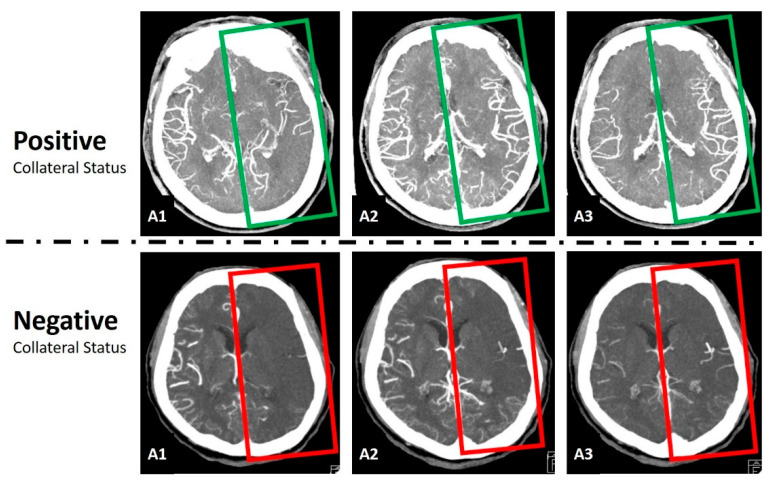
Demonstration of positive and negative collateral status on multiphase CTA. The A1, A2, and A3 indicate the three arterial phases. In the positive collateral status (upper row), the left side brain (green frame) is the ischemic side. When the enhancement of the arterial branches in the ischemic side can achieve similar appearance to the A1 contralateral normal side in A1 or A2 (this case), the collateral status is good or intermediate, respectively, and the result is defined as the positive collateral status. On the contrary, in the negative collateral status (lower row), the arterial branches in the ischemic side (red frame) cannot be enhanced like the A1 contralateral normal side in A1 or A2, suggesting that the collateral status is poor and the result is defined as the negative collateral status.

The collateral status graded as good or intermediate collateral status was defined as the positive collateral result, while poor collateral status as the negative collateral result (Figure 2). From all the enrolled subjects, 70% subjects were randomly selected for the training group, and the remaining 30% of the subjects were placed in the validation group.

**Figure 3:**
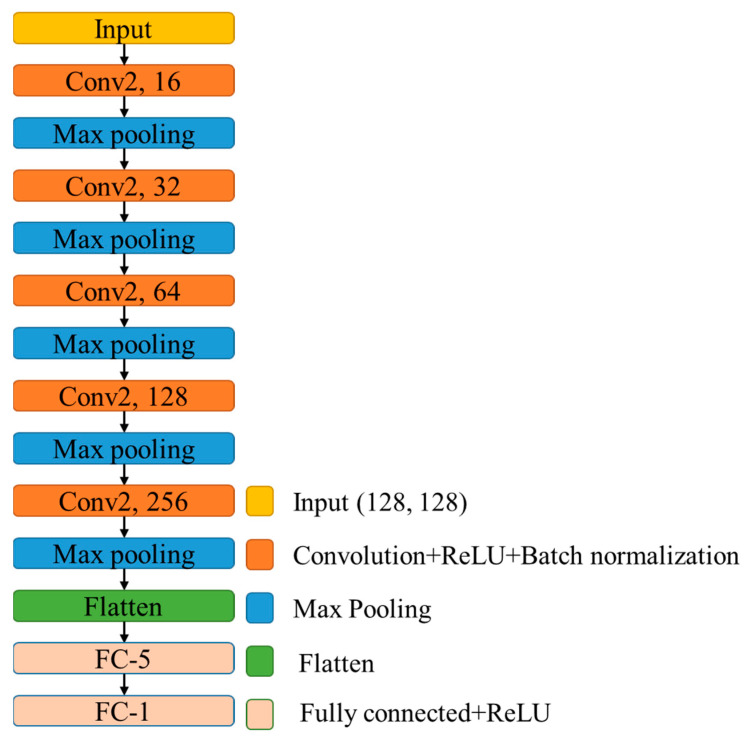
Structure of the CNN model.

The Convolutional Neural Network (CNN) technique was a deep learning technique and was first described in 1980 [12]. This technique was further consolidated by Yann Lecun [13]. CNN could be used to analyze visual imagery with little preprocessing to perform image classification. In this study, the CNN technique was used to develop a prediction model based on the 12 selected mCTA images to predict collateral status as a positive or negative collateral result. Specifically, the structure of the CNN prediction model is shown in Figure 3, where only 5 convolution layers were employed. It was experimentally evaluated to have a comparable performance as that of the renowned ResNet50 [14] trained using transfer learning for this binary classification problem for collateral status, but with a much-improved convergence rate. 

## 3. Results

There were 82 enrolled subjects in this study. The sex ratio was 1:1. The mean age was 70.67 ± 14.09 years old, ranging from 35 to 98 years old. The median of initial NIHSS was 18, ranging from 8 to 31, and the Glasgow Coma Scale was 13.5, ranging from 5 to 15. The median ASPECTS score on the initial nonenhanced brain CT was 8, ranging from 5 to 10. The location of acute arterial occlusion was located at the internal carotid artery in 28 (34.1%) subjects, the first segment of the middle cerebral artery in 41 (50.0%) subjects, and the second segment of the middle cerebral artery in 13 (15.9%) subjects. The procedure results of EVT were 9 (11.0%) with modified treatment in cerebral infarction (mTICI) 0, 4 (4.9%) with mTICI 1, 11 (13.4%) with mTICI 2a, 38 (46.3%) with mTICI 2b, and 20 (24.4%) with mTICI 3. The mean time from onset to reperfusion or end of the procedure was 316.66 ± 86.11 min. Symptomatic intracranial hemorrhage occurred in 14 (17.1%) subjects during the hospitalization course after EVT. The functional outcomes in 3 months were 3 (3.7%) cases with mRS 0, 9 (11.0%) cases with mRS 1, 9 (11.0%) cases with mRS 2, 17 (20.7%) cases with mRS 3, 22 (26.8%) cases with mRS 4, 4 (4.9%) cases with mRS 5, and 18 (22.0%) cases with mRS 6. The demographic data of these subjects with a positive or negative collateral status are separately detailed in Table 1.

**Figure 4:**
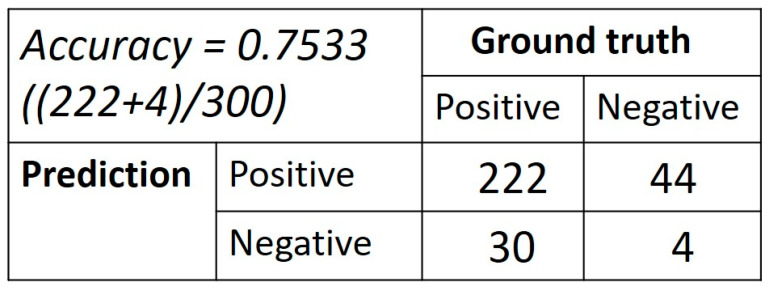
The confusion matrix of the validation group.

**Figure 5:**
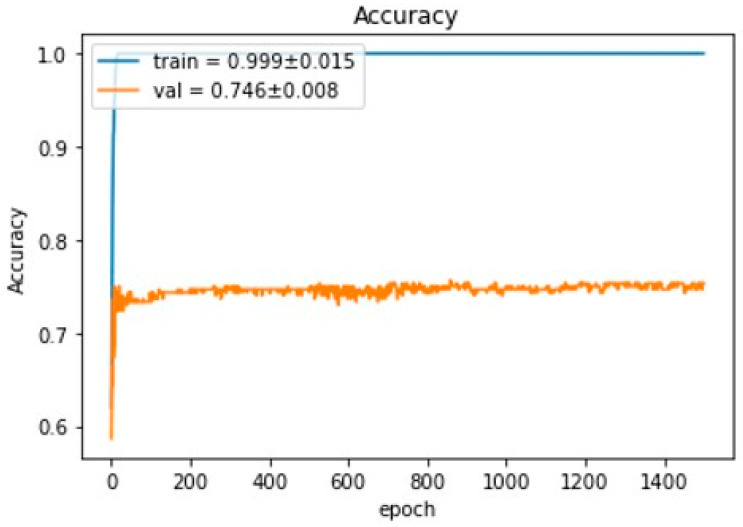
Performance of the prediction model from the selected 12 images of multiphase CTA on the prediction of collateral status.

**Figure 6:**
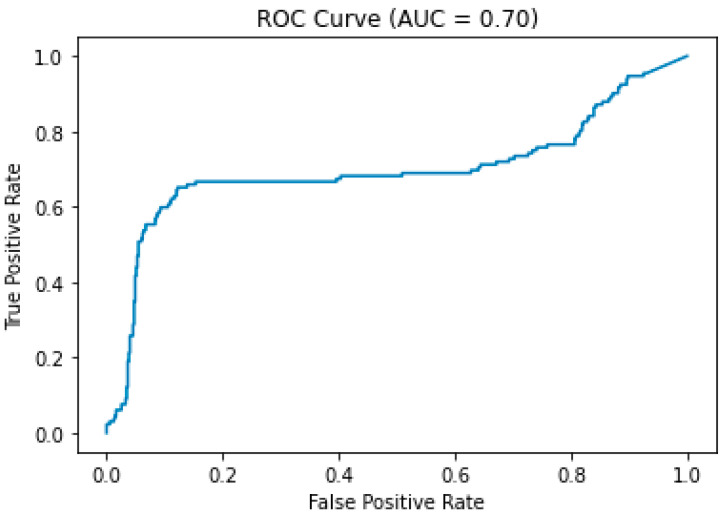
ROC curve of the prediction model with an area under the curve (AUC) of 0.7.

There were 57 cases (69.5%) randomly selected for the training group and 25 cases (30.5%) for the validation group. In the training group, there were 40 cases (70.2%) with a positive collateral result (good or intermediate collateral) and 17 cases (29.8%) with a negative collateral result (poor collateral). In the validation group, there were 21 cases (84.0%) with a positive collateral result and 4 cases (16.0%) with a negative collateral result. The confusion matrix of the validation group is displayed in Figure 4. During training for the CNN prediction model, the accuracy of the training group could reach 0.999 ± 0.015, whereas the prediction model had a performance of 0.746 ± 0.008 accuracy on the validation group (Figure 5). The ROC curve of this model demonstrated an area under the curve (AUC) of 0.7 (Figure 6).

## 4. Discussion

By using the CNN technique to produce a prediction model from the selected 12 images of mCTA, the model demonstrated a performance of 0.746 ± 0.008 accuracy and a 0.7 AUC on the prediction of the collateral status of mCTA.

The collateral status has been found to be an independent predictor of final infarct volume in patients receiving EVT for acute anterior circulation ischemic stroke due to large vessel occlusion. A good collateral status helps to prevent the middle cerebral arterial territory from infarction [15]. On the contrary, poor collateral circulation is a risk factor for symptomatic intracranial hemorrhage after EVT, and therefore results in a poor functional outcome [16,17]. Furthermore, collateral status sometimes might be helpful to determine eligibility for EVT in some cases [18]. There are many proposed methods to evaluate the collateral status by using CT images, including single-phase CTA, mCTA and CTP. The single-phase CTA tends to underestimate the collateral status due to limited information only from the spatial extent of collateral enhancement in one series of images [19]. CTP-based parameters, such as time-to-maximum, cerebral blood flow, hypoperfusion intensity ratio and cerebral blood volume index, have been used to evaluate the collateral status [20,21,22]. By using some automated software, these CTP-based parameters could be calculated automatically and quickly without interobserver bias [19]. However, the availability of an automated software might require an additional expense and several factors including bolus shape, scanner protocol, different software, and head motion might affect CTP analysis [19,23]. In addition, a limited brain coverage of the CT scanner will cause inadequate coverage of the ischemic lesion; therefore, the requirement of a wide-coverage CT scanner is also important to perform a better CTP [24]. On the contrary, collateral evaluation based on mCTA might solve some weak points of CTP-based techniques, but mCTA also has its disadvantages, including reduced temporal resolution and time-consuming visual assessment [19]. Though CTP-based collateral evaluation might be more attractive for clinical practitioners because of faster and fully automatic calculation and more reliable results, mCTA sometimes is the only choice due to the limitation of inaccessible postprocessing software or wide-coverage CT scanners. Recently, the rapid development of AI has significantly contributed to automatic imaging analysis in lesion detection and classification. Thus, the development of automatic analysis on mCTA to predict the collateral status might be possible, and the prediction model would be very useful for hospitals where the mCTA technique is the only choice to evaluate patients with acute ischemic stroke.

A previous study produced an AI model to differentiate good and poor collateral status in mCTA and achieved an accuracy of 0.852 ± 0.045. However, the mCTA was extracted from whole-brain 4D CTA/CTP images rather than the traditional mCTA [25]. Another study used single-phase CTA to produce an AI model and achieved accuracy of 0.85 ± 0.01 to differentiate whether there was more than 50% filling [26]. In an article reviewing recent automatic collateral scoring using CTA, the 4D CTA is the most popular one to be used in the research. Other methods are based on single-phase CTA or even nonenhanced CT [27]. The overall accuracy or area under the curve of these methods could be above 70% or even up to 90%. However, an AI model based on the traditional mCTA to evaluate collateral status is less frequently described in the literature, but our preliminary result suggests that the performance of the AI model based on the traditional mCTA could also achieve more than 70% accuracy with an AUC of 0.7. Based on previous studies, CTP-based ischemic core volume shows a better prediction on the 3-month functional outcome for patients with acute ischemic stroke undergoing EVT than the mCTA collateral score [5]. Fully automated commercial software for CTP analysis are also available. However, CTP requires a higher coverage CT scanner and an additional expense for the commercial software, which sometimes makes CTP an impossible choice even with a CT scanner. The concept of 4D CTA is close to that of the traditional mCTA, though the images of different phases in 4D CTA are extracted from a pool of several volume images, while the images of different phases in the traditional mCTA are directly scanned. Again, 4D CTA also requires a wide-coverage CT scanner. As compared with single-phase CTA and nonenhanced CT, mCTA includes much more imaging information and data. Furthermore, mCTA is found to be highly correlated with the CTP result, and the physiological role might be more important than single-phase CTA [28]. For the abovementioned points, mCTA might be a more reliable resource to produce an AI prediction model for the collateral status because of the similarity to 4D CTA and being more correlated with CTP. Although previous studies mainly adopted 4D CTA, single-phase CTA or nonenhanced CT to produce AI prediction models for the collateral status, our study points out the possibility of automatic evaluation of the collateral status from the traditional mCTA, which might sometimes be the best CT collateral evaluation for acute ischemic stroke in hospitals with limited resources, such as a lack of postprocessing software or wide-coverage CT scanners.

There are several limitations in this study. First, the retrospective design of this study has some diverse settings of scan protocol and many uncontrolled parameters. Second, there are two different CT scanners used in this study, and the heterogeneous quality of images might affect AI analysis. The data only enrolled 82 cases and only included 12 images from each mCTA study. In the future, more enrolled cases and included images might be able to improve the performance of the AI model, but the imaging postprocessing time might increase markedly if more images of mCTA studies are included to evaluate the collateral status. In the confusion matrix of this study, there was a very high false-positive rate (91.7%) and a very low true-negative predictive value (8.3%). Possible reasons for this poor result include limited enrolled subjects and images, relatively more enrolled cases with a positive collateral status, and the complexity of collateral evaluation on mCTA. Because poor collateral status is considered as a negative predictor of functional outcome, cases with poor collateral status are not highly suggested to be aggressively treated with EVT, and, thereby, the cases with poor collateral status are fewer than good or intermediate collateral status in this study. To enroll many more cases with a poor collateral status might be helpful to overcome the problem of a high false-positive rate in the future. There are some challenges we encountered during the AI model training, including the limited number of images, overfitting of models, and poor learning efficiency. There was an important problem about image augmentation we did not expect before this study. Image augmentation is a common technique to create more images from existing ones. Initially, we planned to use image augmentation to overcome the problem of a limited number of images in this study. However, the images of mCTA from a single subject had some internal relationship, because they were from the same subject or from the same scan sequence. When we used image augmentation by flipping or rotating images, the result of model training became worse, probably because of breaking the internal relationship of those images from the same subject. Thus, we were focused on using different AI models or changing the structures of AI models to improve the prediction accuracy.

## 5. Conclusions

This study suggests that even in a hospital with limited resources where only very basic traditional mCTA can be performed, the AI model proposed by this study can still automatically evaluate the collateral status in acute ischemic stroke with large-vessel occlusion, which is promising to facilitate the patient selection for EVT and to decrease inter-observer bias. Since the data required for this AI model only include very basic traditional mCTA images, this AI model can be applied in many more hospitals than other techniques, such as CTP or 4D CTA.

## Figures and Tables

**Table 1:** Demographic data of the enrolled subjects with a positive or negative collateral status.

Collateral Status	Positive (*n* = 61)	Negative (*n* = 21)
	Mean ± SD (Range) *n* (%) Median (Range)	
Age (years)	72.5 ± 13.5 (43–98)	65.4 ± 14.8 (35–96)
Sex (male)	28 (45.9)	13 (61.9)
National Institute of Health Stroke Scale (NIHSS)	17 (8–31)	19 (13–23)
Glasgow Coma Scale	14 (5–15)	11 (7–15)
Alberta Stroke Program Early CT Score (ASPECTS)	8 (5–10)	8 (6–10)
Occlusion site	ICA: 20 (32.8) M1: 31 (50.8) M2: 10 (16.4)	ICA: 8 (38.1) M1: 10 (47.6) M2: 3 (14.3)
Modified treatment in cerebral infarction (mTICI)	0–2a: 16 (26.2) 2b–3: 45 (73.8)	0–2a: 8 (38.1) 2b–3: 13 (61.9)
Onset to Reperfusion time (minutes)	316.3 ± 83.5 (152–528)	317.6 ± 95.4 (189–519)
Symptomatic intracranial hemorrhage	9 (14.8)	5 (23.8)
Modified Rankin Scale	0–2: 18 (29.5) 3–6: 43 (70.5)	0–2: 3 (14.3) 3–6: 18 (85.7)

Abbreviations: ICA: internal carotid artery; M1: the first segment of the middle cerebral artery; M2: the second segment of the middle cerebral artery; SD: standard deviation.

## Data Availability

The data presented in this study are available on reasonable request from the corresponding author.
